# Portomesenteric venous thrombosis post gastric sleeve

**DOI:** 10.1093/jscr/rjac435

**Published:** 2022-09-20

**Authors:** Jaime Ponce-de-León Palomares, Iván González Barajas, Valeria Jaime León, Isaac Esparza Estrada, José A Guzmán Barba, José O Orozco Álvarez-Malo

**Affiliations:** Bariatric Surgery Department, Instituto Nefrológico de Tijuana, Tijuana, Baja California, Mexico; Bariatric Surgery Department, Instituto Nefrológico de Tijuana, Tijuana, Baja California, Mexico; Bariatric Surgery Department, Instituto Nefrológico de Tijuana, Tijuana, Baja California, Mexico; Bariatric Surgery Department, Instituto Nefrológico de Tijuana, Tijuana, Baja California, Mexico; Bariatric Surgery Department, Instituto Nefrológico de Tijuana, Tijuana, Baja California, Mexico; Bariatric Surgery Department, Instituto Nefrológico de Tijuana, Tijuana, Baja California, Mexico

## Abstract

The gastric sleeve is the most performed bariatric surgery, and several studies have shown a good safety profile. Among its main postoperative complications are bleeding, leak, stenosis, reflux and to a lesser extent, portomesenteric venous thrombosis (1%). More than 80% of this entity occur after discharge. Diagnosis is difficult because it does not have characteristic symptoms or laboratory abnormalities. A 30-year-old male with a body mass index of 40.2 kg/m^2^, submitted to gastric sleeve, developing tachycardia, abdominal pain and oral intolerance on the eighth postoperative day. Contrast-enhanced abdominopelvic tomography revealed thrombosis of the portal, mesenteric and splenic veins. Portomesenteric venous thrombosis managed with resection, laparoscopic entero–entero anastomosis and anticoagulation. Although the risk of presenting portomesenteric venous thrombosis is relatively low, its complications are serious and life-threatening, in addition to an increased prevalence in bariatric surgeries.

## INTRODUCTION

Obesity is a chronic and multicausal disease that has reached epidemic proportions throughout the world; The World Health Organization reported that 39% of the adult population were overweight and 13% were obese [1].

Bariatric surgery has proven to be the most effective treatment for obesity. In addition, it achieves a high rate of improvement and remission of metabolic comorbidities [2]. The most widely used techniques for today’s work are based on two mechanisms: restrictive techniques, which reduce gastric capacity to limit food intake due to early satiety, and hypoabsorptive techniques, which exclude a segment of the intestine from contact with nutrients.

Laparoscopic vertical sleeve gastrectomy is the most performed bariatric surgery worldwide [3]. It has mainly a restrictive action, but it also has other effects such as eliminating most of the cells that produce ghrelin [4].

Multiple studies have shown a good safety profile in bariatric surgery, with an estimated mortality of 0.3–0.6% and complications ranging from 2 to 4%, similar to laparoscopic cholecystectomy. Among its main postoperative complications are gastroesophageal reflux (11.7%), stenosis (4.9%), bleeding (1.6%), suture line leakage (0.5–1.5%), deep vein thrombosis (3%) and portomesenteric venous thrombosis (PMVT) (<1%) [5, 6].

## CASE REPORT

A 30-year-old male scheduled for vertical sleeve gastrectomy due to obesity since the age of 15, with a weight of 119 kg and a height of 172 cm (BMI 40.2), without comorbidities or thrombosis family history. Without any previous abdominal surgeries.

An integral preoperative protocol was performed (ASA II, Goldman I, Wells 1; low thromboembolic risk), a 6-day preoperative liquid diet was followed, the patient presented for surgery with a 10% of excess weight loss (%EWL). Laboratory tests were normal.

During surgery, no alterations in the anatomy were identified and no incidents occurred. The procedure lasted 40 min.

In the immediate postoperative period, the patient was asymptomatic, beginning to walk at 4 h and resuming oral feeding with crushed ice at 24 h, vital signs within normal limits, drainage with minimal serohematic output, medicated with ceftriaxone, ketorolac, metamizole sodium and nalbuphine for necessary reason. A water-soluble contrast swallow was performed at 36 h without evidence of leak data. He is discharged due to improvement after 48 h with liquid diet for 1 month, antibiotic treatment (cefuroxime) and analgesic (ketorolac).

**Figure 1:**
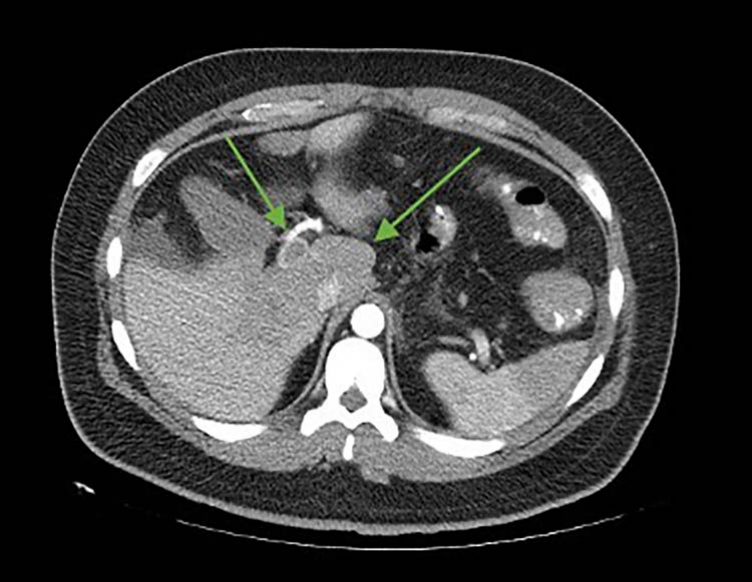
Axial cut. In the arrows, lack of opacification of the portal vein with its hyperdense walls, inferior vena cava, lack of splenic opacity.

**Figure 2:**
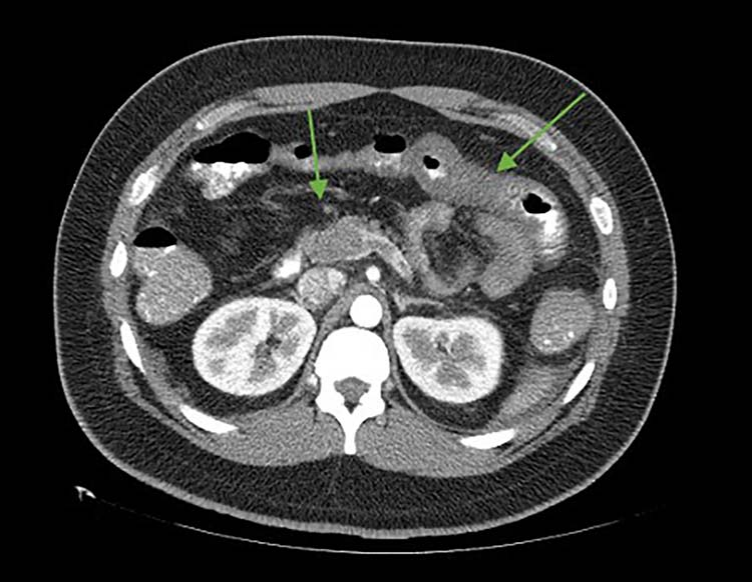
Axial cut. In the arrows, there is a lack of splenomesenteric opacification towards the posterior part of the pancreas and striation of the adjacent peripancreatic fat. Edematous intestinal walls in the jejunum, free fluid in the left paracolic gutter.

On the seventh postoperative day, he began with abdominal pain, tachycardia, fatigue, and anorexia, limited fluid intake and minimal ambulation. On the eighth day, the patient presented to the emergency department due to intolerance to oral feeding, acute abdominal pain (VAS 8/10), in antalgic position, tachycardic and diaphoretic. Physical examination revealed abdominal hyperalgesia and hyperalgesia. Laboratory tests revealed hemoglobin of 11 g/dl, 18 700 leukocytes (95.3% neutrophils), C-reactive protein of 13.3 mg/dl. Blood gases with pH 7.42, lactate 1.6 mmol/L. A contrast-enhanced abdominopelvic tomography showed portal, mesenteric, and splenic vein thrombosis associated with jejunal wall thickening with adjacent mesenteric changes and free fluid in the cavity (Figs 1 and 2).

**Figure 3:**
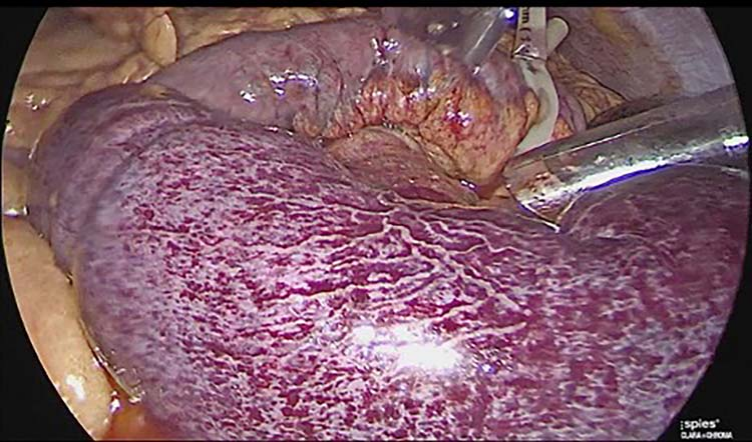
Laparoscopic view of intestinal thrombosis 200 cm at the level of the proximal jejunum, 50 cm from the ligament of Treitz.

**Figure 4:**
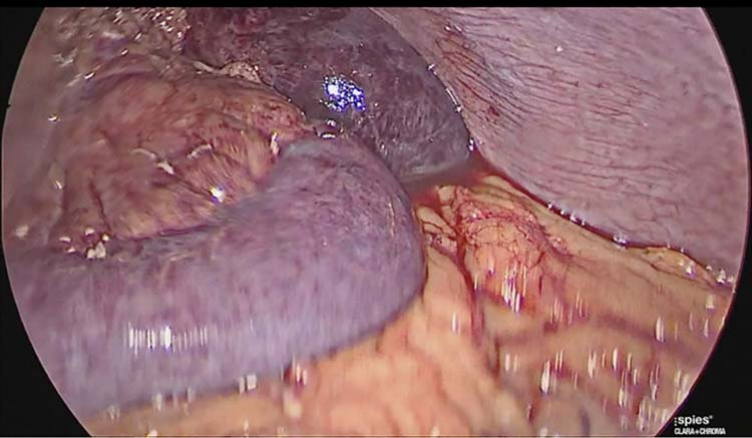
Laparoscopic view of intestinal thrombosis and free fluid in the cavity.

The patient was reoperated by laparoscopy, finding segmental thrombosis from 200 cm to 50 cm of the Treitz ligament, with edema and interloop free fluid without perforation (Figs 3 and 4). Lateral resection and entero–entero anastomosis were performed with manual stapler, subtracting 440 cm of intestine with adequate coloration. Later he was admitted to the Intensive Care Unit, treated with low molecular weight heparin at a full anticoagulation dose, maintaining an INR of 2 and a partial thromboplastin time of 80 s. He presented adequate evolution starting a liquid diet on the fourth postoperative day, new laboratories reported hemoglobin of 11 g/dl, 12 700 leukocytes/mm^3^, platelets 225 000/mcL, INR of 2. He was discharged on the fifth day with rivaroxaban treatment for 6 months.

One year and 4 months after surgery, the patient presented a weight loss of 34.4 kg, currently weighing 84.6 kg and a BMI of 28.6 with a %EWL greater than 50%. He does not take medications on a regular basis and has been maintained only with dietary management and exercise without presenting signs and symptoms of venous thromboembolic disease.

## DISCUSSION

Bariatric surgery procedures are not exempted from complications and can lead to high morbidity and mortality. Obese patients have a higher than average risk of thromboembolic events [7].

As mentioned by Johnson *et al*. [8], PMVT occurs when the thrombus extends into the mesenteric venous system and commonly includes the superior mesenteric vein and the splenic vein. Some risk factors are age, duration of surgery, history of deep vein thrombosis, transfusion, higher BMI, prolonged surgical time and open or revision surgery [9]. Studies suggest that the underlying causes of PMVT after sleeve gastrectomy are: section of the short gastric vessels, aggressive dissection near the pylorus and behind the stomach along the splenic vein, and other general laparoscopic risk factors [10].

More than 80% of post bariatric surgery venous thromboembolism events occur within the first 30 days, the average period described is 15–20 days [11].

The diagnosis requires a high degree of suspicion since it does not have a characteristic clinical or laboratory abnormality, and patients may present a variety of symptoms, including tachycardia, abdominal pain, bloating, absence of bowel movements, nausea, vomiting, dehydration, fever, back pain and even intestinal infarction.

It has been hypothesized that there may be unintentional injury to the gastroepiploic and/or splenic veins that can damage the splanchnic endothelium and lead to local thrombus formation that can then spread throughout the system portal vein [12].

Oral and intravenous contrast-enhanced computed tomography has been reported to diagnose and monitor the patient’s course with a sensitivity of 90% [13].

Wu *et al*. [14] demonstrated that in patients with PMVT detected on images, recanalization with anticoagulation is unlikely and other treatment options such as intestinal resection should be considered.

Some authors recommend extending prophylaxis to 2 weeks in a high-risk patient, also the use of low molecular weight heparin since surgery and for up to 2 weeks after discharge in patients with moderate to severe risk [15] although solid evidence is still needed to update the management and prevention of these complications.
